# Wassermann Test in Relation to Colles’ Law

**Published:** 1914-08

**Authors:** 


**Wassermann Test in Relation to Colles' Law.** J. Cassel of Berlin, Laboratory News, May-June, 1914, gives the following statistics of mothers who had borne syphilitic infants: Cassel, 41 mothers, 25 positive (60%); Knoepfelmacher, 116 mothers, 72 positive (62%); Boas, 81 mothers, 61 positive (75%); F. Lesser, 31 mothers, 23 positive (74%). As the Wassermann test is not absolute and as it may be rendered negative by treatment or, perhaps, by spontaneous cure, the truth of Colles' law can not be definitely determined but the observations are of interest, pending absolute demonstration.

